# Endocrine and Neuroendocrine Tumors Special Issue—Checkpoint Inhibitors for Adrenocortical Carcinoma and Metastatic Pheochromocytoma and Paraganglioma: Do They Work?

**DOI:** 10.3390/cancers14030467

**Published:** 2022-01-18

**Authors:** Camilo Jimenez, Gustavo Armaiz-Pena, Patricia L. M. Dahia, Yang Lu, Rodrigo A. Toledo, Jeena Varghese, Mouhammed Amir Habra

**Affiliations:** 1 Department of Endocrine Neoplasia and Hormonal Disorders, The University of Texas MD Anderson Cancer Center, Houston, TX 77030, USA; jvarghese@mdanderson.org (J.V.); mahabra@mdanderson.org (M.A.H.); 2 Division of Endocrinology, Department Medicine, The University of Texas Health Science Center, San Antonio, TX 78229, USA; armaizpena@uthscsa.edu; 3 Department of Medicine, University of Texas Health San Antonio, San Antonio, TX 78229, USA; dahia@uthscsa.edu; 4 Mays Cancer Center, University of Texas Health San Antonio, San Antonio, TX 78229, USA; 5 Department of Nuclear Medicine, The University of Texas MD Anderson Cancer Center, Houston, TX 77030, USA; ylu10@mdanderson.org; 6 CIBERONC, Gastrointestinal and Endocrine Tumors, Vall d’Hebron Institute of Oncology (VHIO), Centro Cellex, 08035 Barcelona, Spain; rtoledo@vhio.net

**Keywords:** adrenocortical cancer, metastatic pheochromocytoma, metastatic paraganglioma, checkpoint inhibitors, avelumab, ipilimumab, nivolumab, pembrolizumab

## Abstract

**Simple Summary:**

In the past decade, the landscape of cancer treatment has radically changed after the introduction of immunotherapy. Adrenocortical carcinoma and metastatic pheochromocytoma/paraganglioma are rare cancers with limited responses to traditional cancer treatments. The use of immunotherapy against these cancers has yielded a few responses when used alone or in combination with other drugs. We reviewed the current literature to summarize the role of immunotherapy in these rare cancers.

**Abstract:**

Adrenocortical cancers and metastatic pheochromocytomas are the most common malignancies originating in the adrenal glands. Metastatic paragangliomas are extra-adrenal tumors that share similar genetic and molecular profiles with metastatic pheochromocytomas and, subsequently, these tumors are studied together. Adrenocortical cancers and metastatic pheochromocytomas and paragangliomas are orphan diseases with limited therapeutic options worldwide. As in any other cancers, adrenocortical cancers and metastatic pheochromocytomas and paragangliomas avoid the immune system. Hypoxia-pseudohypoxia, activation of the PD-1/PD-L1 pathway, and/or microsatellite instability suggest that immunotherapy with checkpoint inhibitors could be a therapeutic option for patients with these tumors. The results of clinical trials with checkpoint inhibitors for adrenocortical carcinoma or metastatic pheochromocytoma or paraganglioma demonstrate limited benefits; nevertheless, these results also suggest interesting mechanisms that might enhance clinical responses to checkpoint inhibitors. These mechanisms include the normalization of tumor vasculature, modification of the hormonal environment, and vaccination with specific tumor antigens. Combinations of checkpoint inhibitors with classical therapies, such as chemotherapy, tyrosine kinase inhibitors, radiopharmaceuticals, and/or novel therapies, such as vaccines, should be evaluated in clinical trials.

## 1. Introduction

The adrenal glands are very important endocrine organs that are responsible for the regulation of many different physiological mechanisms that preserve human homeostasis and guarantee the individual’s survival. These features include the modulation of the cellular responses to stress; the healing of damaged tissues; the protective responses of fighting and escape; the regulation of the corporal concentrations of acid and electrolytes; the modulation of the metabolism of glucose, fat, and proteins; and the maintenance of adequate blood pressure to satisfy metabolic needs. The adrenal glands regulate homeostasis through the synthesis and secretion of androgens, glucocorticoids, and mineralocorticoids, which are derived from the adrenal cortex, and catecholamines, which come from the adrenal medulla [1,2,3]. Embryologically, the adrenal cortex is derived from the intermediate mesoderm [4], and the adrenal medulla is derived from the neural crest cells close to the dorsal aorta [5]. The adrenal medulla is a modified autonomic sympathetic nervous system ganglion that, unlike other sympathetic ganglia, produces adrenaline and noradrenaline and releases these hormones directly into the bloodstream.

Primary malignant tumors may develop in the adrenal cortex or in the adrenal medulla. The most common cancers to develop in these regions are adrenocortical carcinoma (ACC), which is a tumor derived from the adrenal cortex, and metastatic pheochromocytoma, which is a tumor derived from the adrenal medulla. Similar tumors, called metastatic paragangliomas, develop in the extra-adrenal paraganglia and have genetic and molecular profiles similar to those of many metastatic pheochromocytomas [6], and current clinical trials study metastatic pheochromocytoma and metastatic paragangliomas together. For the purpose of this manuscript, we will consider metastatic pheochromocytomas and paragangliomas (MPPGL) as one tumor group.

ACCs are tumors associated with high proliferative rates and a common clinical phenotype of large, rapidly growing primary tumors that are associated with metastases in up to 80% of cases [7,8,9]. Conversely, pheochromocytomas and paragangliomas are usually characterized by lower proliferative rates than those observed in ACCs, and their metastatic spread is observed in up to 25% of cases [10,11]. Nevertheless, MPPGL tumors are usually large as primary tumors, and the metastases are frequently massive because the diagnosis of these tumors is frequently delayed [9,12]. Most ACCs and MPPGLs secrete excessive amounts of hormones, predisposing patients with these tumors to severe comorbidities. ACCs may secrete large amounts of glucocorticoids, mineralocorticoids, and/or androgens, which may lead to severe Cushing syndrome, hyperaldosteronism, and virilization [13,14], and MPPGLs may secrete excessive amounts of catecholamines, which may lead to severe cardiovascular and gastrointestinal diseases [15,16]. The combination of a large tumor burden, considerable tumor growth over time, and excessive hormonal secretion predisposes patients with ACC and MPPGL to a decreased quality of life and decreased overall survival rates [12,13,14]. In fact, only 15–44% of patients with ACC and 60% of patients with MPPGL are alive 5 years after initial diagnosis [7,17].

ACC and MPPGL are rare tumors. In the United States, approximately 200–300 new cases of ACC and 100–200 new cases of MPPGL are discovered every year [17,18]; by definition, ACC and MPPGL are orphan diseases, and, subsequently, the therapeutic options for advanced disease are limited [10,13]. ACC is mainly treated with a combination of systemic chemotherapy of cisplatin, doxorubicin, etoposide, and mitotane, and clinical responses are noted in approximately 30% of patients; ACC responses to chemotherapy usually have short duration, and treatment toxicity can be substantial [13]. Chemotherapy with cyclophosphamide, vincristine, and dacarbazine for MPPGL is widely available. However, response rates are also low, with approximately 37% of patients achieving partial response (PR), with at least a reduction of 30% of the tumor size when compared with baseline measurements, or disease stabilization; cures are exceptional, and treatment toxicity is also substantial [10,19,20]. Approximately 60–70% of MPPGLs express the noradrenaline transporter; therefore, these tumors are meta-iodine-benzyl-guanidine (MIBG)–avid [12,21]. The United States Food and Drug Administration (FDA) recently approved high-specific-activity MIBG (HSA-I-131-MIBG) for patients with MPPGL. HSA-I-131-MIBG demonstrated a clinical benefit rate (CBR), the proportion of patients who achieve a complete response or tumor disappearance, partial response, and disease stabilization as per RECIST 1.1., higher than 90%, with approximately 25% of patients exhibiting a PR and more than 60% of patients having stable disease with some degree of regression 1 year after treatment. Additionally, most patients who underwent HSA-I-131-MIBG treatment had improved blood pressure compared to baseline, and the toxicity of HSA-I-131-MIBG was acceptable [22]. This medication is only available in the United States and is not indicated for the treatment of patients with MPPGL that does not express the noradrenaline transporter [20].

Given this limited spectrum of therapeutic options, we need to identify other effective treatments for patients with ACC and MPPGL. Over the last decade, immunotherapy with checkpoint inhibitors has become one of the therapeutic pillars against cancer [23]. The results of phase 1 and 2 clinical trials with immunotherapy for ACC and MPPGL have revealed that immunotherapy is a potentially important treatment for patients with these tumors as well. In this review, we will discuss the rationale for the use of immunotherapy against ACC and MPPGL, the results of clinical trials with several checkpoint inhibitors against ACC and MPPGL, and potential mechanisms to induce or enhance an immune system response effective against ACC and MPPGL.

## 2. Avoidance of the Immune System as a Hallmark of Cancer

The hallmarks of cancer are the distinctive and complementary capabilities that enable tumor growth and metastatic dissemination and are the foundation for understanding the biology of any cancer. These hallmarks include the sustaining of the proliferative signaling of tumor cells, mechanisms that favor replicative immortality, genome instability and the presence of mutations, deregulation of cellular energetics and cell necrosis, tumor-promoting inflammation, the induction of abnormal vascular formation or angiogenesis, the activation of mechanisms of invasion and metastases, resistance to tumor cell death, the avoidance of growth suppressors, and the avoidance of the recognition of the cancer cell as such by the immune system [24]. Several features related to cancer cell biology theoretically lead to a destructive anti-cancer immune response. Cancer cells are characterized by the accumulation of a variable number of genetic alterations and the subsequent loss of normal cellular regulatory processes; cancer cells also accumulate neoantigens, antigens of differentiation, and cancer testis antigens, and a fraction of these antigens are bound to major histocompatibility class I molecules, allowing the immune system CD8+ T cells to recognize cancer cells [25,26].

**Figure 1:**
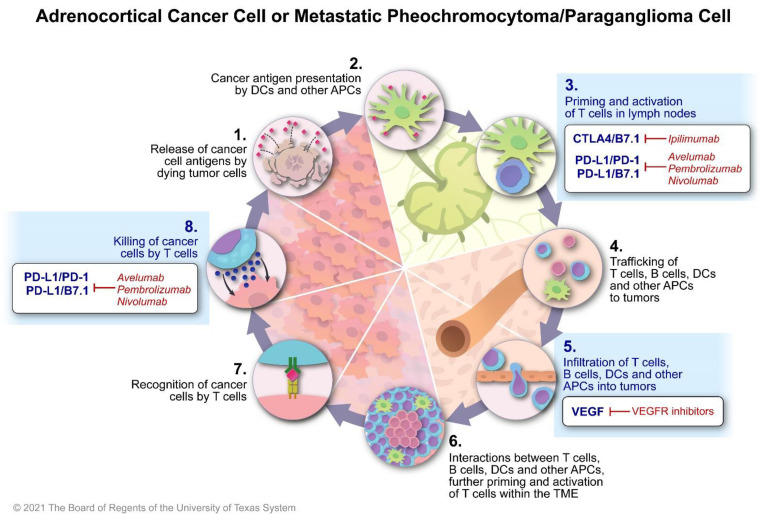
The cancer immunity cycle and the mechanisms of action of checkpoint inhibitors and potential therapies that enhance immune system response. Abbreviations are as follows: PD1: programmed death cell protein 1; PD-L1: programmed death-ligand 1; CTLA-4: cytotoxic T-lymphocyte antigen-4; DC: dendritic cells; APC: antigen presenting cells; VEGF: vascular endothelial growth factor; VEGFR: vascular endothelial growth factor receptor; TME: tumor microenvironment.

Once the cancer cell is recognized as such, the cancer immunity cycle starts [27]. The cancer immunity cycle is a sequence of steps that must be initiated, allowed to proceed, and expanded to generate an anti-cancer immune response (Figure 1) [27]. The cycle starts with the release of antigens that are later presented to antigen-presenting cells, such as the dendritic cells, followed by the priming and activation of T cells in places, such as the lymph nodes. These T cells travel through the bloodstream, identify the location of the tumor cells, infiltrate the tumor environment, and recognize the cancer cells; the T cells then kill the cancer cells with the subsequent release of cancer antigens, enhancing and perpetuating the cancer immunity cycle [27]. The cancer immunity cycle has three important qualities: (1) adaptability, which is the capacity of the immune system to recognize the cancer cell; (2) specificity, which allows the immune system attack to be mainly focused on cancer cells, limiting toxic effects on the normal cells; and (3) memory, which guarantees that the immune system can more effectively recognize and destroy cancer cells that may develop again [27]. Tumors are, however, more than just cancer cells. Tumors are also composed of non-malignant cells that are recruited by the malignant cells to serve them. Together the malignant and non-malignant cells, the extracellular matrix and the tumor vasculature, and their complex communications create the tumor microenvironment.

The cancer immunity cycle is a complex process [27] regulated by many inhibitory or stimulatory factors and pathways in the tumor microenvironment that determine the successful identification of cancer cells by the immune system. While many of these factors have been recognized, many others are unknown. Examples of inhibitory mechanisms of the cancer immunity cycle are the CTLA4 pathway, which prevents antigen-presenting cells from priming and activating T cells; the PD-L1/PD-1 pathway, which prevents the killing of cancer cells; vascular endothelial factors and the endothelin B receptor, which prevent T cells from infiltrating tumors and reduce the expression of the proteins of the major histocompatibility complex by the cancer cells (Figure 1) [27]. Subsequently, several medications that antagonize the activity of inhibitory factors and/or stimulate factors that enhance the cancer immunity cycle have been developed (Figure 1) [23]. Antibodies that block the actions of CTLA4, PD-1, and PD-L1, known as checkpoint inhibitors, such as ipilimumab (CTLA4 inhibitor), pembrolizumab (anti-PD-1 antibody), or nivolumab (anti–PD-L1 antibody), are currently indicated for the treatment of many different malignancies [23]. In some cases, checkpoint inhibitors alone have led to permanent remissions [23]. Antiangiogenic medications, such as cabozantinib, a tyrosine kinase inhibitor, may induce tumor vessel normalization, enhancing tumor infiltration and the recognition of cancer cell by immune T cells [28]. When combined with checkpoint inhibitors, antiangiogenic medications may lead to impressive clinical responses in clear cell renal cell carcinomas [29,30]. Interferon alpha or vaccines made with tumor antigens may facilitate antigen presentation [27]. Clinical trials combining checkpoint inhibitors and vaccines for different types of cancer are ongoing [31]. Chemotherapy, targeted therapies, radiation therapy, and radiopharmaceuticals may release tumor antigens that could be recognized by the immune system. Combinations of these classical systemic therapies with checkpoint inhibitors have been associated with clinical benefits in several malignancies [23,32,33].

In clinical trials, pembrolizumab demonstrated objective responses in patients with advanced clear cell renal cell carcinomas, lung adenocarcinomas, and melanomas that express PD-L1 [34]. Nevertheless, not all tumors that express PD-L1 exhibited objective responses [34]. Many tumors for which pembrolizumab is currently indicated are characterized by an inflamed tumor microenvironment and a high tumor mutation burden (TMB) [35]. ACC may exhibit an inflammatory environment and several somatic mutations [36]; nevertheless, these features are not as notable as the ones noted in the aforementioned cancers [35]. Pheochromocytomas and paragangliomas are associated with minimal or no tumor inflammation and, as such, are classified as “cold” tumors [35]. In addition, MPPGLs are mainly associated with monogenic germline and somatic mutations; in fact, up to 50% of MPPGLs are exclusively associated with germline mutations of the *SDHB* gene, and many MPPGLs have no recognized or a few additional somatic mutations [35]. These observations raise the question of whether immunotherapy with checkpoint inhibitors could be effective for ACC or MPPGL.

## 3. Scientific Rationale for the Potential Use of Checkpoint Inhibitors for ACC or MPPGL

Recent studies have found that some ACCs and MPPGLs express the programmed cell death ligands in tumor cell membranes or stromal cells in the tumor microenvironment [37,38,39]. Small immunohistochemical studies found PD-L1 expression in up to 70% of ACC samples [38] and 18% of MPPGL samples [37], suggesting that several patients with ACC and some patients with MPPGL may benefit from checkpoint inhibitors, such as avelumab, nivolumab, or pembrolizumab. In addition, it has been recognized that 3–18% of ACC cases are associated with somatic or germline mutations of DNA mismatch repair genes, such as *ML1*, *MSH2*, *MSH6*, and *PMS2* mutations (e.g., ACC-associated Lynch syndrome), and these mutations lead to microsatellite instability [40,41,42]. The use of checkpoint inhibitors seems to be an appealing option for this subset of ACC cases. In fact, the FDA approved the use of pembrolizumab for the treatment of any solid cancer associated with DNA mismatch repair gene mutations and microsatellite instability [43]. This approval was based on an impressive overall response rate (ORR) of pembrolizumab of approximately 40% (including some complete responses), with a duration of benefits longer than 6 months in 78% of patients treated with pembrolizumab across five clinical trials for several malignancies [43]. Nevertheless, these trials did not include ACC patients, so checkpoint inhibitors for ACC with DNA mismatch repair gene mutations and microsatellite instability must be evaluated through clinical trials. Importantly, microsatellite instability is associated with an increased TMB in approximately 4% of ACC [44].

In general, the cancer microenvironment is characterized by a rapid proliferation of the tumor cells that is unmatched by the available blood supply. To compensate, abnormal vessels develop; however, the supply of oxygen is still limited leading to hypoxia with subsequent stabilization of the inducible (alpha) subunit of hypoxia inducible factors (HIFs) [45]. HIFs activate the PD-L1 gene, which, in turn, induces tumor immune escape by suppressing the activity of cytolytic T cells [45]. Most MPPGL are characterized by an environment of pseudohypoxia [46]. Up to 50% of patients with MPPGL carry germline mutations of the subunit B of the succinate dehydrogenase gene (*SDHB*), and metastatic tumors also happen in carriers of other mutations involved in the regulation of the oxygen metabolism (e.g., *SDHA*, *SDHC*, *SDHD*, *FH*, and *VHL* genes) [46]. Furthermore, many apparently sporadic MPPGs are characterized by a microenvironment of pseudohypoxia, including those that carry activating mutations of *EPAS1*, the gene encoding for HIF2 alpha [46,47]. Therefore, it is worth exploring checkpoint inhibitors for patients with MPPGL. Of interest, there are rare cases of succinyl dehydrogenase gene mutation–associated ACC [48]. However, because these cases are very rare, it is unlikely that pseudohypoxia is a major player determining a potential response to checkpoint inhibitors in ACC.

For a variety of reasons, checkpoint inhibitors are a potentially attractive therapy to orphan tumors [49]. Unlike other systemic therapies, immunotherapy with checkpoint inhibitors has been demonstrated as an effective treatment for many different cancers, irrespective of their embryological origin and histological characteristics [23]. In addition, clinical responses do not seem to always correlate with PD-L1 expression in the tumor cells and/or tumor microenvironment [50]. Adverse events associated with checkpoint inhibitors are, for the most part, acceptable and correctable with supportive measures [23,49,51]. Furthermore, there are no reliable preclinical models to predict the actions of immunotherapy in specific malignancies. The following sections describe the results of phase 1 and 2 clinical trials against ACC and MPPGL.

## 4. Clinical Trials with Immune Checkpoint Inhibitors

### 4.1. Clinical Trials with Immune Checkpoint Inhibitors for ACC

#### 4.1.1. Avelumab

Avelumab, a PD-L1 inhibitor, was the first checkpoint inhibitor evaluated for ACC in clinical trials. Avelumab’s pharmacokinetics, efficacy, and safety were evaluated in JAVELIN, a phase 1b international, multicenter clinical trial that included 50 patients with progressive metastatic ACC [52]. Objective responses (PR) were seen in 3 patients (6%), but the CBR was 48%. Almost half of the study participants received concomitant mitotane therapy, including two of the three patients with PR. A large majority of ACCs progressed over a short time; thus, the median progression-free survival (PFS) was only 2.6 months, and overall survival (OS) was 10.6 months [52]. Toxicity was acceptable, although 16% of patients had grade ≥3 adverse events. The study showed that 60% of tumors did not express PD-L1, and these patients exhibited shorter PFS and OS when compared with patients with PD-L1–positive tumors. Nevertheless, this difference was not statistically significant. The few PRs did not correlate with PD-L1 expression [52].

#### 4.1.2. Nivolumab

Nivolumab is a PD-1 inhibitor that was evaluated in a phase 2 investigator-initiated, single-center clinical trial for patients with ACC [53]. The primary endpoint of this trial was ORR. This small trial included 10 patients with progressive ACC who were either previously treated with platinum-based chemotherapy and/or mitotane or who were therapy naïve. The results of the study indicated that nivolumab did not elicit a response. ACC progressed in seven patients. Two patients had stable disease, one for a very short period and the other for 48 weeks. One patient had an unconfirmed PR; however, this patient withdrew from the trial because of a severe side effect. As expected, toxicity was acceptable overall and similar to what has been observed in clinical trials for nivolumab treatment of other malignancies. Finally, the PFS was only 1.8 months [53].

#### 4.1.3. Pembrolizumab

Like nivolumab, pembrolizumab is a PD-1 inhibitor. Pembrolizumab has been evaluated in two phase 2 clinical trials for patients with ACC. The first published phase 2 clinical trial with pembrolizumab included 16 patients with advanced ACC [54,55]. These patients previously underwent failed standard systemic therapy for ACC. The primary endpoint was the non-progression rate at 27 weeks. Two patients were not evaluable for the primary endpoint. Five patients (36%) did not have disease progression 27 weeks after treatment was initiated. The ORR was 14%, and the CBR was 57%. Tumor responses did not correlate with PD-L1 tumor expression, tumor-infiltrating lymphocytes, microsatellite instability, or tumor hormonal activity [55]. In fact, none of the patients had PD-L1 expression. Nevertheless, clinical observations suggest that clinical responses were more likely in patients with tumors that did not secrete hormones when compared with those with tumors associated with Cushing syndrome [55]. Nevertheless, tumors associated with Cushing syndrome achieved either PR or stable disease, suggesting that combining pembrolizumab with medications that lower cortisol production may lead to better responses [55]. Pembrolizumab had severe side effects, including colitis and pneumonitis.

The second trial with pembrolizumab evaluated ORR as the primary endpoint and included 39 patients [56]. The ORR was 39%, and the CBR was 52%. However, the median PFS was only 2.1 months, and the median OS was 24.9 months. Serious adverse events were noted in 13% of patients. Positive tumor responses did not correlate with PD-L1 expression or microsatellite instability. This study did not provide information on tumor hormonal activity [56].

#### 4.1.4. Ipilimumab Plus Nivolumab

In a phase 2 clinical trial of ipilimumab plus nivolumab for patients with rare genitourinary tumors, a subset of 16 patients with advanced ACC was included. The ORR was only 6%; however, the CBR was almost 50%. The toxicity of ipilimumab plus nivolumab was acceptable [57]. The results of this study suggest that the combination of these two checkpoint inhibitors do not provide better responses than what is noticed in patients with ACC treated with single-agent pembrolizumab.

#### 4.1.5. Does Immunotherapy with Checkpoint Inhibitors Work for ACC?

In general, the ORRs for single-agent immune checkpoint inhibitors are low; however, the CBRs for these inhibitors are more impressive. Almost half of the patients treated with these therapies have shown at least disease stabilization, which is frequently associated with some degree of tumor regression for some time; in addition, the risk for significant toxicity of these inhibitors is much lower when compared with that of platinum-based chemotherapy. Furthermore, the few patients who respond to checkpoint inhibitors often enjoy a long duration of response [55,56], and a few cases of complete responses have been seen outside the clinical trials [58]. Nevertheless, it is currently very difficult to predict which patients may benefit from immunotherapy. Clinical trials have demonstrated that the expression of PD-L1 in ACC does not necessarily predict a positive clinical outcome; in fact, there are some patients with very impressive radiographic responses with ACC samples lacking PD-L1 expression [52,55]. Conversely, there are patients with PD-L1 expression in whom antitumor responses are not observed [55]. Although the absence of PD-L1 expression could represent a mechanism of tumor resistance to checkpoint inhibitors, observations from clinical trials indicate that this process is much more complicated.

However, other mechanisms of avoidance of the immune system have been proposed [59]. These mechanisms may include, but are likely not limited to, the frequently observed inactivation of the *P53* gene pathway in ACC due to somatic and occasional germline mutations of *P53* (as are common with Li-Fraumeni syndrome) [36,60]. These mutations may lead to the decreased recruitment of natural killer and other immune cells [61,62,63] or the sometimes-noted upregulation of the WNT/β-catenin pathway [36,60], which may impair adequate antigen presentation, chemotaxis, and tumor infiltration by T cells [59,64]. Nevertheless, clinical trials have not explored correlations with these molecular phenotypes, and these phenotypes may not necessarily predict an immunotherapy response. Moreover, ACC linked to excessive glucocorticoid secretion is associated with worse prognosis when compared with non-hormonally active ACC [7]. The glucocorticoid-related toxicity suggests an increased risk for complications, such as osteoporosis and fractures, muscle weakness, hypertension, and especially immune suppression, which may predispose patients to systemic infections. Furthermore, ACC associated with Cushing syndrome exhibits elevated mitotic rates and, subsequently, more aggressive oncological behavior; these characteristics, together with the inherent comorbidity of Cushing syndrome, lead to lower overall survival rates compared with non-hormonally active tumors [36].

Considering the modest mutation burden of ACC, it is speculated that the effective immune targeting of ACC will require combination therapy or an engineered cellular therapy [65]. Emerging data show that checkpoint inhibitors combined with other treatments may overcome the mechanisms of avoidance or resistance to the immune system. Based on the concern that excess cortisol creates an unfavorable atmosphere for immunotherapy [63], a phase 1b study is ongoing to evaluate the effect of combining pembrolizumab with relacorilant, a glucocorticoid receptor blocker (ClinicalTrials.gov Identifier: NCT04373265). In addition, checkpoint inhibitors may be combined with inhibitors of adrenal glucocorticoid synthesis, such as metyrapone.

The combination of mitotane, as the standard of care, with immune checkpoint inhibitors has been reported with avelumab, and this combination is likely safe, considering the relatively low risk of severe adverse events in the study [52]. A recent retrospective case series of six patients treated with pembrolizumab and mitotane found durable responses in the majority of patients, including two patients with a durable response rate [66]. This combination is of great interest because it can utilize the adrenolytic and steroid reduction properties of mitotane to make the tumors more susceptible to checkpoint inhibitor therapy.

In the past few years, the use of antiangiogenic agents has transformed the management of multiple malignancies. Targeting vascular endothelial growth factor receptor signaling, in combination with checkpoint inhibitors, e.g., by combining pembrolizumab and lenvatinib in renal cell carcinoma and endometrial carcinoma, has proven beneficial in multiple malignancies and resulted in exceptionally high response rates [67,68]. It is hypothesized that combining immunotherapy with antiangiogenic drugs has synergistic effects that enhance response rates [69]. A recent cases series evaluated the combination of lenvatinib with pembrolizumab in ACC. Despite undergoing many failed lines of therapy, some patients treated with this combination had durable responses to therapy, whereas checkpoint inhibitors and antiangiogenic therapy as single-agent therapies were unsuccessful [70].

### 4.2. Clinical Trials with Immune Checkpoint Inhibitors for MPPGL

#### 4.2.1. Pembrolizumab

A phase 2 clinical trial with pembrolizumab at 200 mg intravenously every 3 weeks explored the actions of PD-1 inhibition against MPPGL [71]. The primary endpoint of this trial was a non-progression rate at 27 weeks (9 cycles) greater than 20%, based on the Response Evaluation Criteria in Solid Tumors 1.1. Secondary endpoints included ORR, CBR, PFS, OS, safety, and correlations with PD-L1 expression and infiltrating mononuclear inflammatory cells in the primary tumor with disease response and genotype. The clinical trial included 11 patients with progressive MPPGL. Sixty-four percent of patients had apparently sporadic MPPGL, 18% had paraganglioma syndrome type 4 (germline *SDHB* mutations), 1 patient had paraganglioma syndrome type 1 (germline *SDHD* mutation), and 1 patient had a germline *PMS2* mutation. Sixty-four percent of patients had tumors that secreted noradrenaline. Fifty-five percent of the primary tumors were in the sympathetic extra-adrenal paraganglia, 36% of patients had pheochromocytomas, and 1 patient had a primary head and neck paraganglioma. Patients had an acceptable performance status, with an Eastern Cooperative Oncology Group performance score of ≤1. Only 28% of patients were naïve to therapy; most patients had previously undergone cyclophosphamide, vincristine, and dacarbazine chemotherapy, and treatment with HSA-I-131-MIBG, and/or tyrosine kinase inhibitors. Forty percent of patients had no evidence of disease progression at 27 weeks; however, the ORR was only 9%. The toxicity of pembrolizumab was acceptable, and there were no grade 4 or 5 side effects or cases of catecholamine crisis. The median PFS was 5.7 months, and the median OS was 19 months, with 55% of patients deceased at the time of publication of clinical trial results because of tumor progression [71]. There was no clear association between PD-L1 expression or tumor-infiltrating lymphocytes in the primary tumor with clinical response, genetic background, or hormonal activity.

**Figure 2:**
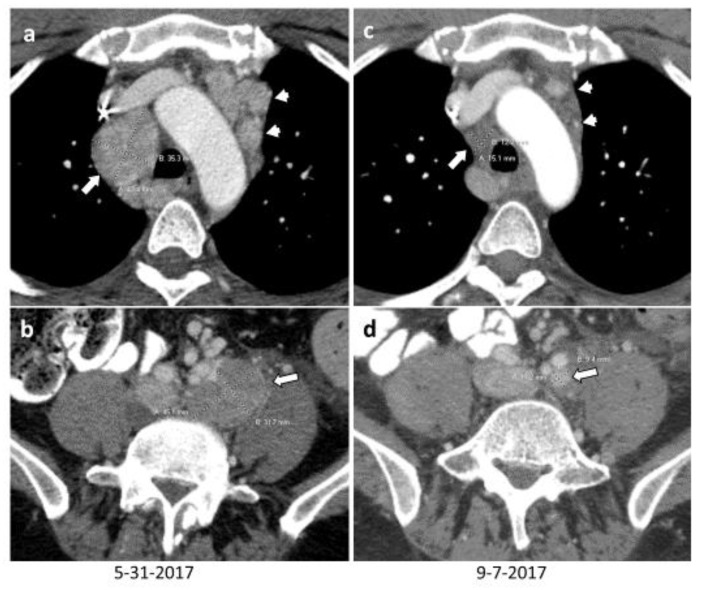
A 42-year-old man with metastatic paraganglioma underwent multiple surgery resection, post-surgical therapy with CVD × 6 months, cabozantinib × 8 months, then treatment with pembrolizumab. The pre-immunotherapy contrast enhanced CT (CECT) showed innumerable lymph node metastases in the chest, abdomen, and pelvis. At 2 months post initiation of immunotherapy, the lymph node metastases had significantly decreased by number and size (long and short arrows in images (**a**–**d**)). Representative axial CECT images ((**a**): pretherapy axial CECT of chest, (**b**): pretherapy axial CECT of pelvis, (**c**): post-therapy axial CECT of chest, (**d**): post-therapy axial CECT of pelvis) showed that the mediastinal (long and short arrows in (**a**,**c**)) and left common iliac (long arrow in (**b**,**d**)) lymph node metastases had significantly improved. The right paratracheal lymph node (long arrows in (**a**,**c**)) decreased from 4.3 × 3.5 cm to 1.5 × 1.2 cm, and the left common iliac lymph node (long arrows in (**b**,**d**)) decreased from 4.5 × 3.2 cm to 1.1 × 0.9 cm.

A clinical assessment indicated that only two patients (18%) had an obvious benefit. One patient had a non-hormonally active tumor that achieved a confirmed immune-related partial response that persisted for longer than 2 years. The patient with the most impressive clinical response had paraganglioma syndrome type 4 metastatic paraganglioma associated with excessive noradrenaline secretion, overwhelming symptoms of catecholamine excess, and massive lymph node, lung, liver, and skeletal metastases. The patient had previously undergone cabozantinib treatment, which was complicated by severe hand and foot syndrome and a superinfection with pseudomonas aeruginosa. Cabozantinib was then discontinued, and the patient exhibited a rapid progression. Several metastases were palpable and visible on physical examination. The blood pressure was difficult to control, and the patient complained of palpitation, sweats, and headaches. The patient underwent pembrolizumab treatment. Four days after infusion, the metastases were no longer palpable or visible, the blood pressure normalized, and several antihypertensives were discontinued, as the patient complained of near syncopal episodes. Symptoms of catecholamine excess were no longer reported. Radiographic studies found a 56% tumor size reduction (Figure 2). The patient had elevated levels of liver enzymes, which delayed treatment with pembrolizumab. At the time of radiographic follow-up three months later, a new liver lesion was noted, and the patient discontinued his participation in the trial [71].

#### 4.2.2. Ipilimumab Plus Nivolumab

This phase 2 clinical trial included two patients with progressive MPPGL [57]. One patient did not experience a response to the therapy and exhibited disease progression. The other patient had stable disease for longer than 2 years, with excellent performance status, occasional fatigue, and no symptoms of tumor burden. However, the tumor size did not decrease [57]. Whether the second patient exhibited disease stabilization because of immunotherapy or because of the nature of MPPGL tumors, which may become stable with no intervention despite initial growth, is still to be defined.

#### 4.2.3. Does Immunotherapy with Checkpoint Inhibitors Work for MPPGL?

The authors of the manuscript and others believe that checkpoint inhibitors and other types of immunotherapy can work for patients with MPPGL [72]. However, we need to better understand the mechanisms that may successfully activate the immune system. At this time, the clinical scientific experience with checkpoint inhibitors is limited to the definitive results of the small phase 2 clinical trial with single-agent pembrolizumab and the limited results of a phase 2 clinical trial that combined nivolumab and ipilimumab [71,72]. The results of these trials indicate that checkpoint inhibitors are associated with modest responses and that pembrolizumab alone or ipilimumab combined with nivolumab should not be considered as first-line therapies for patients with MPPGL [71,72]. Combining checkpoint inhibitors with other therapeutic modalities that enhance antigen recognition and/or facilitate vascular normalization could activate the immune system more successfully. Preliminary results of a phase 2 clinical trial with cabozantinib (the most potent antiangiogenic medication available in clinical practice) seem impressive and suggest that a substantial number of patients with MPPGL may benefit from this medication [28]. Similarly, a recent report described a patient who had a positive oncological response characterized by tumor size reduction and stabilization after receiving 40 mg of cabozantinib daily for 7.5 months. The patient was then treated with pembrolizumab with no response, followed by chemotherapy, which caused substantial toxicity. The patient later underwent a combined treatment of cabozantinib with nivolumab. Combining cabozantinib with nivolumab was associated with a PR, disease stabilization, the disappearance of the symptoms of catecholamine excess, and an acceptable toxicity, and the clinical benefits of this combined therapy lasted for 22 months. This case report suggests that cabozantinib could have induced some degree of tumor vascular normalization that facilitated the activation of the immune system [73].

In the phase 2 clinical trial with pembrolizumab, one patient had an impressive clinical response, raising the question of whether previous exposure to cabozantinib and/or the introduction of foreign bacterial antigens can activate the immune system against MPPGL. Pseudomonas aeruginosa is a Gram-negative bacterium, characterized by the presence of lipopolysaccharides in the outer layer and many other components that trigger or enhance an immune response. These bacteria may become trapped in the tumor microenvironment within the abnormal vessels, inducing an immune attack against tumor cells, thus supporting bacteria-based MPPGL immunotherapy [74,75].

A recent retrospective study found that patients with MPPGL may have a higher incidence of other malignancies, such as lung, prostate, melanoma, and colorectal cancers, when compared to the general population [76]; this finding suggests that some of the MPPGL tumorigenesis pathways and mechanisms of immune resistance could be similar to the ones observed in more common tumors for which immunotherapy has been demonstrated to be effective; thus, learning from the experience with the aforementioned malignancies may provide clues on how to treat MPPGL with immunotherapies.

## 5. The Gut Microbiome and Peptide-Based Vaccination against ACC and MPPGL

Peptide-based vaccination delivers immunogenic peptides, corresponding to tumor-associated or tumor-specific antigens, to elicit a T-cell immune response. It is challenging to generate a strong immune response against tumor-associated antigens (TAAs), mainly because the non-mutated tumor-associated antigens are part of the repertoire of self-antigens. To circumvent this problem, the immune response should target mutated, non-self-antigens.

Sequencing of the human fecal microbiota revealed that all TAAs had a closely structurally related “mimic” in the microbiome, with higher affinities for the MHC than the corresponding TAA [77]. As these “mimics” are produced by bacteria, they have the potential to “pre-expose” any person and generate memory T-cells; the re-activation and subsequent expansion of these T cells can generate a robust response against the TAAs. The links between the microbiome, clinical response, and inhibition of cancer progression in cancer patients treated with targeted immunotherapies or with specific chemotherapeutic agents have already been emphasized [78]. The presence of commensal bacteria-specific memory T cells in the gut and in the periphery has been described as well [79]. These microbiome-derived peptides stimulate strong immune responses against TAAs and trigger in vivo tumor regression after vaccination.

The NCT04187404 trial (SPENCER Trial) is evaluating the vaccine EO2401 against ACC and MPPGL. This vaccine includes three microbiome-derived CD8+ epitopes mimicking parts of TAAs, such as the interleukin receptor alpha 2 (IL13Rα2), survivin (BIRC5), and the mammalian forkhead box M1 (FOXM1); these antigens are overexpressed and linked to clinical outcomes in ACC and MPPGL [80,81,82,83,84,85,86,87]. These antigens may induce an immune response against tumors of adrenal origin and have minimal to no expression in normal organs. The SPENCER trial is a multicenter, phase 1/2, first-in-human study to assess the safety, tolerability, immunogenicity, and preliminary efficacy of EO2401 in combination with nivolumab for patients with untreated or previously treated ACC or MPPGL. The initial data from the trial is awaited in 2022.

## 6. Conclusions

Immunotherapy for adrenal tumors, such as ACC and MPPGL, is at an early stage of development. Single-agent immunotherapy has led to some impressive and durable responses in patients with ACC; however, ORRs are generally low. In patients with MPPGL, responses have been uncommon, and the mechanisms of response are unclear. However, the failure of checkpoint inhibitors to elicit a response is not an indication of an absolute lack of success. Conversely, the failure of single-agent checkpoint inhibitor therapy represents an opportunity to identify the mechanisms that could lead to more successful treatment strategies. Combining checkpoint inhibitors with chemotherapy, mitotane, tyrosine kinase inhibitors, and/or vaccines for ACC or chemotherapy, tyrosine kinase inhibitors, and/or radiopharmaceuticals for MPPGL needs to be proactively explored.
